# Supplementary figures and images for: Effectiveness of nutritional countermeasures in microgravity and its ground-based analogues to ameliorate musculoskeletal and cardiopulmonary deconditioning–A Systematic Review
Source: PLoS One. 2020 Jun 9;15(6):e0234412. doi: 10.1371/journal.pone.0234412 (PMC7282646; doi:10.1371/journal.pone.0234412)

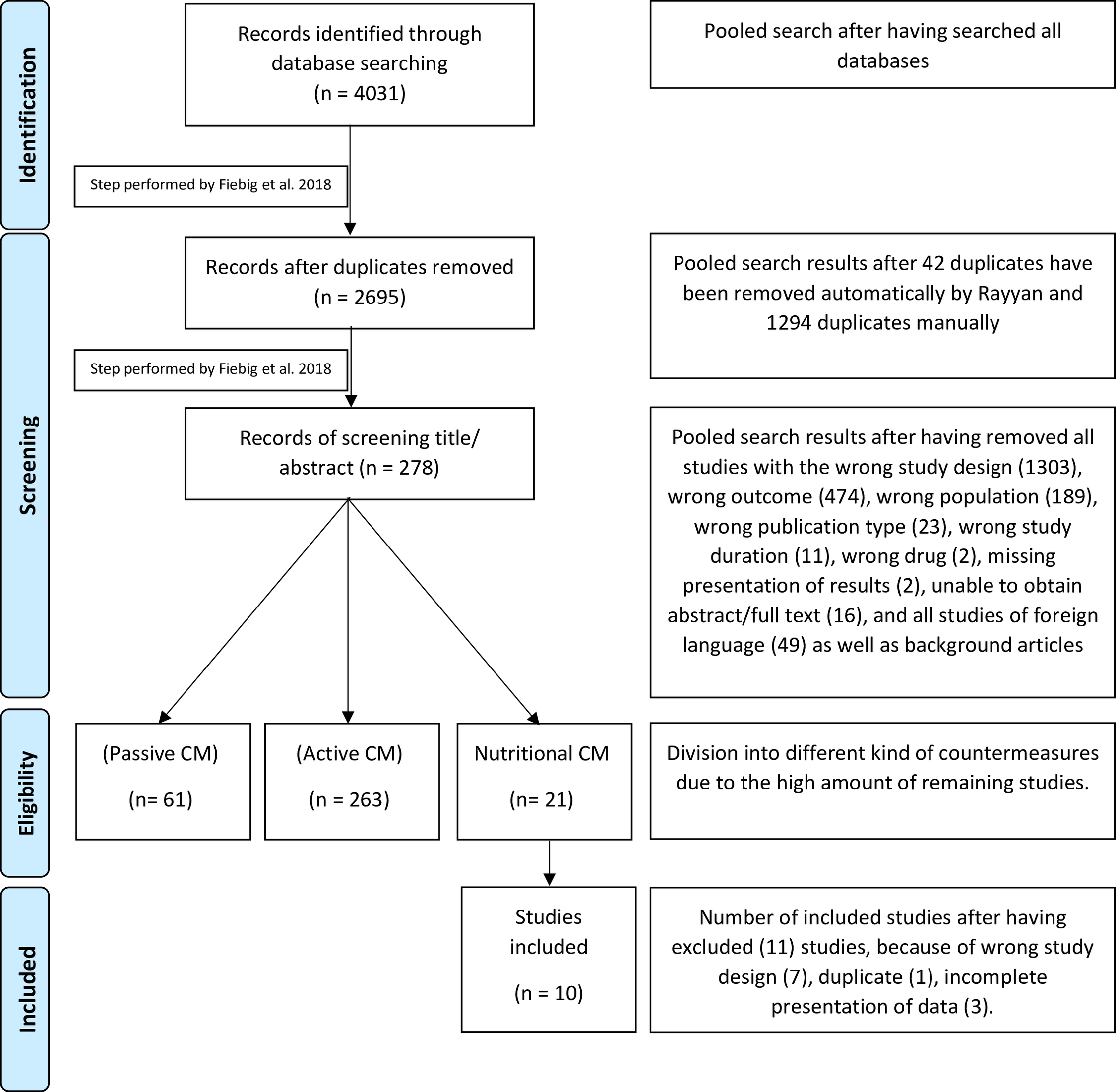

Supplement: S1 Fig — Initial literature search and screening procedure performed by Leonie et al. 2018. Literature screening was performed using Rayyan web application. CM = countermeasure. (Figure adapted from Fiebig et al. [35]). (TIF) [file pone.0234412.s001.tif]
