# Supplementary material for: Effectiveness of nutritional countermeasures in microgravity and its ground-based analogues to ameliorate musculoskeletal and cardiopulmonary deconditioning–A Systematic Review
Source: PLoS One. 2020 Jun 9;15(6):e0234412. doi: 10.1371/journal.pone.0234412 (PMC7282646; doi:10.1371/journal.pone.0234412)
Supplement: S1 Table — (DOCX) [file pone.0234412.s002.docx]

**Study characteristics**

| **Author + year** | **BR days** | **Population** | **Design** | **Intervention** | **Outcome measures** | **Applied test** | **Mean difference CG (±SD)** | **Mean difference IG (±SD)** |  |
| --- | --- | --- | --- | --- | --- | --- | --- | --- | --- |
| **Arbeille et al. 2012** | 60 | 16 | RCT | LPD | Number of finishers | Tilt + LBNP test after BR | NA | NA |  |
| **Beller et al. 2011** | 60 | 16 | RCT | LPD | Muscle CSA [cm^2^] | Radius 66% | -1.18 ± 6.10 | -1.07 ± 6.15 |  |
| **Beller et al. 2011** | 60 | 16 | RCT | LPD | Muscle CSA [cm^2^] | Tibia 66% | -12.39 ± 13.50 | -10.02 ±13.61 |  |
| **Beller et al. 2011** | 60 | 16 | RCT | LPD | BMD [mg/cm^2^] | Radius 4% total, | -0.25 ± 39.62 | 2.14 ± 39.16 |  |
| **Beller et al. 2011** | 60 | 16 | RCT | LPD | BMD [mg/cm^2^] | Radius 4% trabecular | -2.84 ± 32.60 | -0.90 ± 32.32 |  |
| **Beller et al. 2011** | 60 | 16 | RCT | LPD | BMD [mg/cm^2^] | Radius 66% cortical | -1.20 ± 41.10 | 4.51 ± 41.41 |  |
| **Beller et al. 2011** | 60 | 16 | RCT | LPD | BMD [mg/cm^2^] | Tibia 4% total | -9.30 ± 35.80 | -9.36 ± 34.47 |  |
| **Beller et al. 2011** | 60 | 16 | RCT | LPD | BMD [mg/cm^2^] | Tibia 4% trabecular | -6.22 ± 32.90 | -6.97 ± 32.35 |  |
| **Beller et al. 2011** | 60 | 16 | RCT | LPD | BMD [mg/cm^2^] | Tibia 66% cortical | -0.12 ± 39.6 | -2.65 ± 39.51 |  |
| **Beller et al. 2011** | 60 | 16 | RCT | LPD | BMD [mg/cm^2^] | Total hip | -0.031 ± 0.95 | -0.037 ± 0.98 |  |
| **Beller et al. 2011** | 60 | 16 | RCT | LPD | BMD [mg/cm^2^] | Total lumbar | 0.0044 ± 0.99 | 0.016 ± 0.99 |  |
| **Beller et al. 2011** | 60 | 16 | RCT | LPD | BMD [mg/cm^2^] | Whole-body: total | -0.003 ± 1.06 | -0.011 ±1.07 |  |
| **Beller et al. 2011** | 60 | 16 | RCT | LPD | BMD [mg/cm^2^] | Whole-body: head | 0.0038 ± 1.56 | 0.0007 ± 1.51 |  |
| **Beller et al. 2011** | 60 | 16 | RCT | LPD | BMD [mg/cm^2^] | Whole-body: arm | 0.0083 ± 0.86 | 0.0089 ± 0.87 |  |
| **Beller et al. 2011** | 60 | 16 | RCT | LPD | BMD [mg/cm^2^] | Whole-body: trunk | -0.015 ± 0.95 | -0.018 ± 0.95 |  |
| **Beller et al. 2011** | 60 | 16 | RCT | LPD | BMD [mg/cm^2^] | Whole-body: leg | -0.014 ± 1.06 | -0.020 ± 1.09 |  |
| **Bosutti et al. 2016** | 21 | 10 | RCT | Protein + KHCO_3_ | Torque [nM] | Max knee extension | -52 ± 16 | -63 ± 15 |  |
| **Bosutti et al. 2016** | 21 | 10 | RCT | Protein + KHCO_3_ | Torque [nM] | Max plantar flexion | -29 ± 12 | -19 ± 13 |  |
| **Bosutti et al. 2016** | 21 | 10 | RCT | Protein + KHCO_3_ | VO_2_ max [L/min] | NA | -0.52 ± 0.42 | -0.60 ± 0.42 |  |
| **Bosutti et al. 2016** | 21 | 10 | RCT | Protein + KHCO_3_ | VO_2_ max [L/min/kg] | NA | -0.0066 ± 0.01 | -0.0074 ± 0.02 |  |
| **Florian et al. 2015** | 14 | 9 | RCT | Normal energy intake | Number of finishers | LBNP test after BR | NA | NA |  |
| **Lee et al. 2014** | 60 | 16 | RCT | LPD | Peak torque [Nm] | Knee extension | -34.78 ± 5.2 | -16.36 ± 26.9 |  |
| **Lee et al. 2014** | 60 | 16 | RCT | LPD | Peak torque [Nm] | Plantar flexion | -29.83 ± 8.6 | -18.98 ± 13.8 |  |
| **Lee et al. 2014** | 60 | 16 | RCT | LPD | Peak torque [Nm] | Dorsi flexion | -3.37 ± 1.7 | -4.29 ± 2.6 |  |
| **Lee et al. 2014** | 60 | 16 | RCT | LPD | Total work [J] | Knee extension | -145.4 ± 66.6 | -222.7 ± 120.3 |  |
| **Lee et al. 2014** | 60 | 16 | RCT | LPD | Strenght [kg] | Leg press | -12.3 ± 5.95 | -20.88 ± 8.56 |  |
| **Rejc et al. 2015** | 35 | 19 | CT | High energy intake | Max explosive power [W] | Lower limbs | -839 ± 246 | -801 ± 199 |  |
| **Schneider et al. 2007** | 60 | 16 | RCT | LPD | Heart rate [bpm] | Supine | 11.61 ± 3.26 | 10.78 ± 1.94 |  |
| **Schneider et al. 2007** | 60 | 16 | RCT | LPD | Heart rate [bpm] | Standing | 52.39 ± 3.19 | 55.99 ± 2.45 |  |
| **S. Trappe et al. 2008** | 60 | 15 | RCT | LPD | Peak force [mN] | MHC I | -0.2 ± 0.52 | -0.17 ± 0.067 |  |
| **S. Trappe et al. 2008** | 60 | 15 | RCT | LPD | Peak force [mN] | MHC IIa | -0.14 ± 0.07 | -0.27 ± 0.13 |  |
| **S. Trappe et al. 2008** | 60 | 15 | RCT | LPD | Peak power [un*FL-1*s-1] | MHC I | -2.5 ± 0.89 | -2.8 ± 1.08 |  |
| **S. Trappe et al. 2008** | 60 | 15 | RCT | LPD | Peak power [un*FL-1*s-1] | MHC IIa | -2.8 ± 5.19 | -15.2 ± 9.91 |  |
| **S. Trappe et al. 2008** | 60 | 15 | RCT | LPD | Norm. power [W/L] | MHC I | -0.16 ± 0.14 | -0.05 ± 0.34 |  |
| **S. Trappe et al. 2008** | 60 | 15 | RCT | LPD | Norm. power [W/L] | MHC IIa | 0.8 ± 0.92 | -0.9 ± 1.70 |  |
| **S. Trappe et al. 2008** | 60 | 15 | RCT | LPD | Po/CSA [kN/m^3^] | MHC l | -12 ± 6.65 | -2 ± 7.81 |  |
| **S. Trappe et al. 2008** | 60 | 15 | RCT | LPD | Po/CSA [kN/m^3^] | MHC Ila | -2 ± 7.61 | -9 ± 11.83 |  |
| **S. Trappe et al. 2008** | 60 | 15 | RCT | LPD | Diameter [um] | MHC I | -13 ± 5 | -12 ± 3.61 |  |
| **S. Trappe et al. 2008** | 60 | 15 | RCT | LPD | Diameter [um] | MHC Ila | -9 ± 2.83 | -11 ± 7.21 |  |
| **T.A Trappe et al. 2007** | 60 | 16 | RCT | LPD | Isometric force [N] | Supine squat | -681 ± 154 | -780 ± 265 |  |
| **T.A Trappe et al. 2007** | 60 | 16 | RCT | LPD | Concentric peak force [N] | Supine squat | -228 ± 79 | -327 ± 116 |  |
| **T.A Trappe et al. 2007** | 60 | 16 | RCT | LPD | Eccentric peak force [N] | Supine squat | -232 ± 104 | -293 ± 98 |  |
| **T.A Trappe et al. 2007** | 60 | 16 | RCT | LPD | Isometric force [N] | Calf press | -923 ± 214 | -1157 ± 341 |  |
| **T.A Trappe et al. 2007** | 60 | 16 | RCT | LPD | Concentric peak force [N] | Calf press | -665 ± 246 | -673 ± 214 |  |
| **T.A Trappe et al. 2007** | 60 | 16 | RCT | LPD | Eccentric peak force [N] | Calf press | -584 ± 255 | -684 ± 219 |  |
| **T.A Trappe et al. 2007** | 60 | 16 | RCT | LPD | Concentric peak power [W] | Supine squat | -105 ± 33 | -165 ± 56 |  |
| **T.A Trappe et al. 2007** | 60 | 16 | RCT | LPD | Concentric peak power [W] | Calf press | -164 ± 44 | -183 ± 50 |  |
| **T.A Trappe et al. 2007** | 60 | 16 | RCT | LPD | Work [J] | Calf press | 19.11 ± 19 | -73 ± 21 |  |
| **T.A Trappe et al. 2007** | 60 | 16 | RCT | LPD | Work [J] | Supine squat | -56 ± 21 | -99 ± 36 |  |
| **T.A Trappe et al. 2007** | 60 | 16 | RCT | LPD | Muscle volume [cm^3^] | Quadriceps femoris | -152 ± 50 | -170 ± 62 |  |
| **T.A Trappe et al. 2007** | 60 | 16 | RCT | LPD | Muscle volume [cm^3^] | Triceps Surae | -108 ± 18 | -104 ± 46 |  |
| **Zwart et al. 2005** | 28 | 13 | RCT | Amino acids + carbohydrates | BMC [g/cm^2^] | Total body | 10 ± 188 | -31 ± 211 |  |

Study characteristics sorted by author presenting number of BR days, study population, study design, intervention, outcome measures, applied test, mean differences (±SD) from pre- to post bed rest in the control group (CG) and the nutritional intervention group (IG). BMC = bone mineral content; BMD = bone mineral density; BR = bed rest; CSA = cross sectional area; CT = controlled trial; LBNP = lower body negative pressure; LPD = leucine protein diet; Max = maximum; MHC = myosin heavy chain; NA = not applicable; Norm. power = peak power normalized to cell size; P_o_ = peak force; RCT = randomized controlled trial; VO_2_ = volume oxygen.
