# Supplementary material for: Effectiveness of nutritional countermeasures in microgravity and its ground-based analogues to ameliorate musculoskeletal and cardiopulmonary deconditioning–A Systematic Review
Source: PLoS One. 2020 Jun 9;15(6):e0234412. doi: 10.1371/journal.pone.0234412 (PMC7282646; doi:10.1371/journal.pone.0234412)
Supplement: S1 File — (DOCX) [file pone.0234412.s004.docx]

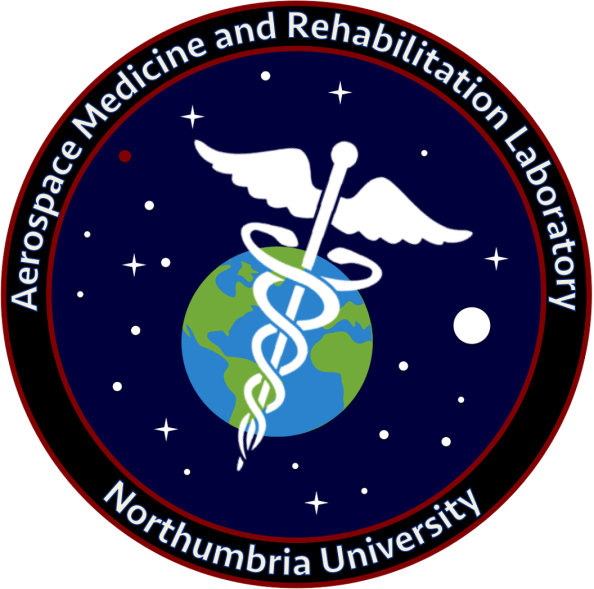


**Protocol Template**

**Aerospace Medicine Systematic Review Group**

**Protocol**

**Review Title**

Effectiveness of nutritional countermeasures to prevent cardiopulmonary and musculoskeletal deconditioning in astronauts and healthy participants of spaceflight analogue studies – A Systematic Review

**Review Information**

Peter H. Sandal, Leonie Fiebig, Nick Caplan, Andrew Winnard, David Green, Tobias Weber

Dr Tobias Weber

KBRwyle for ESA – European Space Agency

EAC - European Astronaut Centre

Linder Höhe, D-51147 Cologne, Germany

[tobias.weber@esa.int](mailto:tobias.weber@esa.int)

**Background**

**Description of condition:**

The condition of interest is deconditioning of the cardiopulmonary and musculoskeletal systems during spaceflight and spaceflight analogues such as bed rest and dry immersion.

**Description of populations:**

The main population of interest is astronauts. However, due to the expected lack of high quality studies in that population, healthy men and women exposed to spaceflight analogues with high transferability such as bed rest and dry-immersion will be included. The control group will be healthy men and women exposed to microgravity, bed rest and dry-immersion without receiving nutritional countermeasure interventions.

**Description of interventions:**

All forms of nutritional countermeasure interventions will be included. Nutritional countermeasures are nutritional supplements administered in addition to a regular diet or a special diet prescribed to affect the physiological parameters of interest.

**How interventions might work:**

Nutritional supplements affect several physiological systems, including the cardiovascular and musculoskeletal systems, by reducing the adverse effects on cardiovascular parameters such as orthostatic tolerance and musculoskeletal parameters such as bone mineral density.

**Why it is important to do this review:**

Effective nutritional countermeasures are needed to prevent cardiovascular and musculoskeletal decondition during space flight to preserve astronauts’ health and performance during and after space flights. On future long duration exploration class space vehicles there will be limited space for heavy equipment such as resistive exercise devices. It is, therefore, of great importance to optimize nutritional countermeasures in order to complement and, if possible, replace equipment dependent countermeasures. In addition, there is a need to optimize the time consumption of in-mission exercise to allow astronauts to perform more important tasks. However, there is a lack of knowledge on the effectiveness of nutritional countermeasures to preserve the cardiovascular and musculoskeletal systems during space flights and the currently applied nutritional countermeasure regimen are not fully evidence-based.

**Aim and Objectives**

This systematic review aims to identify the most effective nutritional countermeasures for the prevention of cardiopulmonary and musculoskeletal deconditioning in order to guide recommendations of in-mission nutritional regimens and future directions of research in nutritional countermeasures for long duration space flight.

It’s objectives include:

1. Evaluate the effectiveness of nutritional countermeasures to prevent cardiopulmonary and musculoskeletal deconditioning during spaceflight
2. Identify the most effective nutritional countermeasures regimen to complement and possibly replace countermeasures dependent on space-occupying equipment and reduce exercise time in space
3. Guide directions of future research on nutritional countermeasures for long duration space flight.

**Methods**

**Criteria for considering studies for this review**

- **P**opulation: Astronauts (space missions), bed rest and dry-immersion participants
- **I**nterventions: Supplemental countermeasures
- **C**ontrol conditions: Participants exposed to microgravity, bed rest or dry-immersion who did not receive any form of (nutritional) countermeasure interventions.
- **O**utcomes:
- Bone:
  1. Bone mineral density (BMD)
  2. Bone mineral content (BMC)
  3. Markers:
     - - 1. CTX
         2. XPTX
         3. XOC (Osteocalcin)
- Muscle:
  1. Muscle volume
  2. Muscle mass
  3. Muscle thickness
  4. Muscle cross sectional area (physiological and/or anatomical)
  5. Muscle force
  6. Joint moments
- Cardiopulmonary:
  1. Cardiac output
  2. Stroke volume
  3. Heart rate
  4. Blood pressure (systolic/diastolic)
  5. Blood volume
  6. Plasma volume
  7. Orthostatic tolerance
  8. Maximal oxygen uptake (VO2max)
  9. Aerobic/Anaerobic threshold
  10. Resting heart rate
- **S**tudy designs: Randomised controlled trials (RCT), controlled clinical trials (CT) and before and after studies.

**Electronic searching**

A range of keywords, grouped by main search terms, were used in various combinations (Boolean logic) to search the following databases for English language articles: PubMed, Web of Science, Cochrane Collaboration Library, Institute of Electrical and Electronics Engineers database as well as ESA’s ‘Erasmus Experiment Archive’, the National Aeronautics and Space Administration’s (NASA) ‘Life Science Data Archive’ and ‘Technical Reports Server’ and the German Aerospace Centre’s (DLR) database

| **Main category** | **Specific category** | | **Keywords in Boolean search format** | **Search number** | **Search mask** |
| --- | --- | --- | --- | --- | --- |
| Microgravity | Synonyms | | "space analogue" OR "ground-based analogue" OR "terrestrial analogue" OR "space flight" OR space-flight OR spaceflight OR "Space mission" OR "space station" OR “micro gravity” OR micro-gravity OR microgravity OR spaceflight OR weightless* OR "orbital flight" OR "zero gravity" OR "space shuttle" | 1 | Abstract/ Title |
|  | Methods & simulations | | "bed rest" OR bed-rest OR "dry immersion" OR dry-immersion | 2 | Abstract/ Title |
|  |  | | #1 AND #2 | 3 |  |
|  | Population of interest | | Astronaut* OR astronaut [Mesh] OR cosmonaut* OR taikonaut* | 4 | Abstract/ Title |
|  |  | | #1 OR #3 OR #4 | 5 |  |
| Countermeasures | Active countermeasures | | Countermeasure* OR exercis* OR exercise [Mesh] OR sport* OR "physical activity" OR "physically active" | 6 | All Fields |
|  | Passive countermeasures | | Centrifug* OR suit* OR "lower body negative pressure" OR LBNP or "fluid loading" OR garment OR stimulation OR "artificial gravity" OR "axial loading" OR electromyostimulation OR "electrical muscle stimulation" OR EMS OR "neuromuscular electrical stimulation" OR NMES OR "whole body vibration" OR WBV | 7 | All Fields |
|  | Nutritional countermeasures | | Diet, food, and nutrition [Mesh] OR nutrition* OR diet* OR food* OR supplement* OR protein* OR salt OR saline OR bi-phosphonate OR phosphonate OR nucleotide* OR vitamin* | 8 | All Fields |
|  |  | | #6 OR #7 OR #8 | 9 |  |
| Relevant parameters for mission safety | Cardiopulmonary & -vascular | Physical performance | "endurance" OR Vo2 OR Vo2max OR Vo2peak OR "maximal oxygen uptake" OR "peak oxygen uptake" OR "resting heart rate" OR "peak power" OR "maximal work load" OR "orthostatic tolerance" OR "orthostatic intolerance" OR "time until presyncope" OR "exercise tolerance" OR "central fatigue" OR "threshold" OR "onset of blood lactate accumulation" OR "OBLA" | 10 | All Fields |
|  | Biomechanical | Physical performance | "muscle strength" OR "muscular strength" OR "muscle function" OR "muscular function" OR "muscle power" OR "muscular power" OR "muscle force" OR "muscular force" OR fatigability OR "fatigue resistance" OR "peripheral fatigue" OR "joint moment" OR "joint moments" OR "postural stability" OR posture OR "postural control" OR balance OR sway OR motion OR locomotion OR gait OR walk* OR run* OR jump* OR hop* OR "movement quality" OR "movement pattern" OR "motion pattern" OR coordination OR "motor control" OR "core stability" OR "core strength" OR "trunk stability" OR "trunk strength" OR "lumbopelvic stability" OR "lumbo-pelvic stability" OR "lumbopelvic control" OR "lumbo-pelvic control" | 11 | All Fields |
|  |  | Anthropometrics | Anthropometr* OR "skeletal strength" OR "bone mineral density" OR "bone density" OR "bone mineral content" OR flexib* OR "range of movement" OR "range of motion" | 12 | All Fields |
|  |  |  | #10 OR #11 OR #12 | 13 |  |
|  |  |  | #5 AND #9 AND #13 | 14 |  |
|  |  |  | Apply human filter |  |  |

**Data collection and analysis**

- Inclusion:
- All forms of random controlled trials, controlled trials and before and after studies with healthy men and women exposed to microgravity, bed rest or dry-immersion and nutritional countermeasure interventions with a no intervention or placebo control group.
- Exclusion:
- Studies using modelled data
- Animal studies
- Studies with ill/disabled subjects
- Any space flight analogue other than bed rest or dry immersion.
- Three independent reviewers will perform the inclusion/exclusion screening. Rayyan software will be used to guarantee a blinded screening process.
- Quality screening will be performed applying Cochrane’s risk of bias procedures and the AMSRG tool for assessment of bed rest quality.
- Data extraction will be performed using the AMSRG data extraction forms. A qualitative analysis will be followed by a quantitative (effect sizes/meta analysis) provided the quality of extracted data allows to do so.

**Contribution of authors**

**Peter H. Sandal:** Acquisition of data, analysis and/or interpretation of data; drafting the manuscript, critical revision of the article, final approval of the version to be published

**Leonie Fiebig**: Conception and design of study, acquisition of data, critical revision of the article, final approval of the version to be published

**Nick Caplan**: Methodological guidance, Acquisition of data, analysis and/or interpretation of data; drafting the manuscript, critical revision of the article, final approval of the version to be published

**Andrew Winnard:** Methodological guidance, critical revision of the article, final approval of the version to be published

**David Green**: Scientific guidance, critical revision of the article, final approval of the version to be published

**Tobias Weber**: Conception and design of study, scientific guidance, acquisition of data, analysis and/or interpretation of data; drafting the manuscript, critical revision of the article, final approval of the version to be published
