# Supplementary material for: Effectiveness of nutritional countermeasures in microgravity and its ground-based analogues to ameliorate musculoskeletal and cardiopulmonary deconditioning–A Systematic Review
Source: PLoS One. 2020 Jun 9;15(6):e0234412. doi: 10.1371/journal.pone.0234412 (PMC7282646; doi:10.1371/journal.pone.0234412)
Supplement: Attachment — Submitted filename: Response to Reviewers.docx [file pone.0234412.s005.docx]

18/05/2020

Subject: Rebuttal Letter for PONE-D-20-10013

Dear Dr Reddy,

Thank you very much for your email. We have highly appreciated the helpful comments and suggestions from you and the reviewer and fully considered them in the revised manuscript. In order to facilitate the debate, we are addressing the comments point by point. Please find the reviewers’ and your comments highlighted in red color, followed by our answers in black color. We hope these changes will meet the requirements to be further considered for publication in *PLOS ONE*.

We have also amended the funding statement and declare herewith that the funder (KBR GmbH) provided support in the form of salaries for authors [DAG, TW], but did not have any additional role in the study design, data collection and analysis, decision to publish, or preparation of the manuscript. The specific roles of these authors are articulated in the ‘author contributions’ section. The affiliations of the authors DAG and TW do not alter our adherence to PLOS ONE policies on sharing data and materials.

Kind regards,

Tobias Weber

**Comments from Dr Sakamuri V. Reddy:**

Though the authors have conducted an extensive survey of nutritional countermeasures to  musculoskeletal and cardiopulmonary challenges in microgravity conditions, I suggest improving  the quality of all figures, details in all legends including the  supplemental information provided.

Thank you. We have done as suggested.

**Comments from Dr Prof Banerjee:**

Dear Professor Banerjee,

Thank you for your helpful suggestions and comments. Please find your comments highlighted in red color, followed by our answers in black color below. All changes in the revised manuscript have been highlighted using the track changes function in word.

In this manuscript, Dr Weber and colleagues performed a systematic review to evaluate the effectiveness of nutritional countermeasure to ameliorate the physiological challenges of the musculoskeletal and cardiopulmonary systems in microgravity conditions necessary to maintain human health during space flight missions. The survey concluded that nutrition alone cannot be expected to be an effective countermeasure in maintaining musculoskeletal and cardiopulmonary integrity during space flight and bed rest. The authors have performed systematic review with a thorough background literature. Specific comments to further improve the manuscript are as follow.

- Thank you for your thorough review!

Title- The authors may consider rephrasing the title for a review article- “Nutritional countermeasures to musculoskeletal and cardiopulmonary challenges in microgravity conditions”.

- Thanks for proposing a new title. However, the authors of the present manuscript feel that the original title better reflects study design and purpose of the study, so we decided to keep the title as it is.

They have conducted a Systematic Review and Meta-Analyses of data. They have used PRISMA 2009 flow diagram. I suggest the authors to verify the journal policy and using statistical methods appropriately.

- Thank you for highlighting this, and apologies if this was not clear. We have not done a full meta-analysis due to the high heterogeneity of study designs and outcomes. More precisely, we did not do a meta-analysis, as either the RCT/CT data was too heterogeneous, or there were only within participant designs which would break the meta analysis statistical assumptions. However, to still provide an overall summary, we converted all included data to effect sizes in standardised units to give an overall comparable summary of the evidence base as it stands today. In conclusion, we have used statistical methods appropriately and the present methodology is in line with the highest standards available for systematic literature reviews (Cochrane guidelines). We have added a sentence to the data analysis section to make this clear.

Also, improve the quality of all figures provided. Fig.1 legend, please provide ref#35 for Fiebig et al 2018 noted.

- Thank you. We have done as suggested.

References: Please remove the repeated information for references provided ex., Ref#21: “Available from:----------”

- Thank you. We have done as suggested.

Verify complete details for Ref#37 “AMSRG Data extraction form v2. 2017” following a journal article for citations listed.

- Thank you. We have verified this and revised the references in the bibliography.

Although the Methods section include Data analysis, I suggest providing details in legends with for Figures 1-4 with statistical methods and significance data (p.15); also, details in legends for supporting information data provided.

- Thank you. We have done as suggested

This is a very elaborate and detailed review regarding the effect of microgravity on the health of Astronauts during prolonged space flight. The authors have analyzed with effective statistical tools published data from several papers related to this field and have reported the results of their analysis on bone density, cardiovascular health, renal, vitamins, minerals, orthostatic hypotension,immune system and other relevant physiological changes pertaining to disturbance of normal homeostasis during prolonged space flight. This is a very well researched manuscript and could be a great source for information for biologist and physiologists doing microgravity research.

- Once again, thank you for your thorough review!

**Journal requirements:**

When submitting your revision, we need you to address these additional requirements.

 1. Please ensure that your manuscript meets PLOS ONE's style requirements, including those for file naming. The PLOS ONE style templates can be found at

<https://journals.plos.org/plosone/s/file?id=wjVg/PLOSOne_formatting_sample_main_body.pdf> and

<https://journals.plos.org/plosone/s/file?id=ba62/PLOSOne_formatting_sample_title_authors_affiliations.pdf>

- Thank you. We verified this and can confirm that the PLOSONE requirements are met.

2. Please provide the date range for when the literature was searched.

- Thank you. We have done as suggested and added this information to the methods section.

3. Thank you for stating the following in the Financial Disclosure section:

'The author(s) received no specific funding for this work.'

We note that one or more of the authors are employed by a commercial company: KBR GmbH

 a. Please provide an amended Funding Statement declaring this commercial affiliation, as well as a statement regarding the Role of Funders in your study. If the funding organization did not play a role in the study design, data collection and analysis, decision to publish, or preparation of the manuscript and only provided financial support in the form of authors' salaries and/or research materials, please review your statements relating to the author contributions, and ensure you have specifically and accurately indicated the role(s) that these authors had in your study. You can update author roles in the Author Contributions section of the online submission form.

Please also include the following statement within your amended Funding Statement.

“The funder provided support in the form of salaries for authors [insert relevant initials], but did not have any additional role in the study design, data collection and analysis, decision to publish, or preparation of the manuscript. The specific roles of these authors are articulated in the ‘author contributions’ section.”

- Thank you. We have done as suggested and added this information to the author contribution section.

If your commercial affiliation did play a role in your study, please state and explain this role within your updated Funding Statement.

- Not applicable.

b. Please also provide an updated Competing Interests Statement declaring this commercial affiliation along with any other relevant declarations relating to employment, consultancy, patents, products in development, or marketed products, etc.

- Not applicable.

Within your Competing Interests Statement, please confirm that this commercial affiliation does not alter your adherence to all PLOS ONE policies on sharing data and materials by including the following statement: "This does not alter our adherence to  PLOS ONE policies on sharing data and materials.” (as detailed online in our guide for authors [http://journals.plos.org/plosone/s/competing-interests](about:blank)) . If this adherence statement is not accurate and  there are restrictions on sharing of data and/or materials, please state these. Please note that we cannot proceed with consideration of your article until this information has been declared.

- We have done as suggested and added this information to the author contribution section.

c. Please include both an updated Funding Statement and Competing Interests Statement in your cover letter. We will change the online submission form on your behalf.

- We have done as suggested.

Please know it is PLOS ONE policy for corresponding authors to declare, on behalf of all authors, all potential competing interests for the purposes of transparency. PLOS defines a competing interest as anything that interferes with, or could reasonably be perceived as interfering with, the full and objective presentation, peer review, editorial decision-making, or publication of research or non-research articles submitted to one of the journals. Competing interests can be financial or non-financial, professional, or personal. Competing interests can arise in relationship to an organization or another person. Please follow this link to our website for more details on competing interests: [http://journals.plos.org/plosone/s/competing-interests](about:blank)

- This has been noted, and the PLOS ONE information on competing interests has been reviewed, and the corresponding author has declared all potential conflicts of interest on behalf of all contributing authors.

While revising your submission, please upload your figure files to the Preflight Analysis and Conversion Engine (PACE) digital diagnostic tool, <https://pacev2.apexcovantage.com/>. PACE helps ensure that figures meet PLOS requirements. To use PACE, you must first register as a user. Registration is free. Then, login and navigate to the UPLOAD tab, where you will find detailed instructions on how to use the tool. If you encounter any issues or have any questions when using PACE, please email us at [figures@plos.org](mailto:figures@plos.org). Please note that Supporting Information files do not need this step.

- We have done as suggested.
